# Supplementary figures and images for: PS-SiZer map to investigate significant features of body-weight profile changes in HIV infected patients in the IeDEA Collaboration
Source: PLoS One. 2020 May 1;15(5):e0220165. doi: 10.1371/journal.pone.0220165 (PMC7194369; doi:10.1371/journal.pone.0220165)

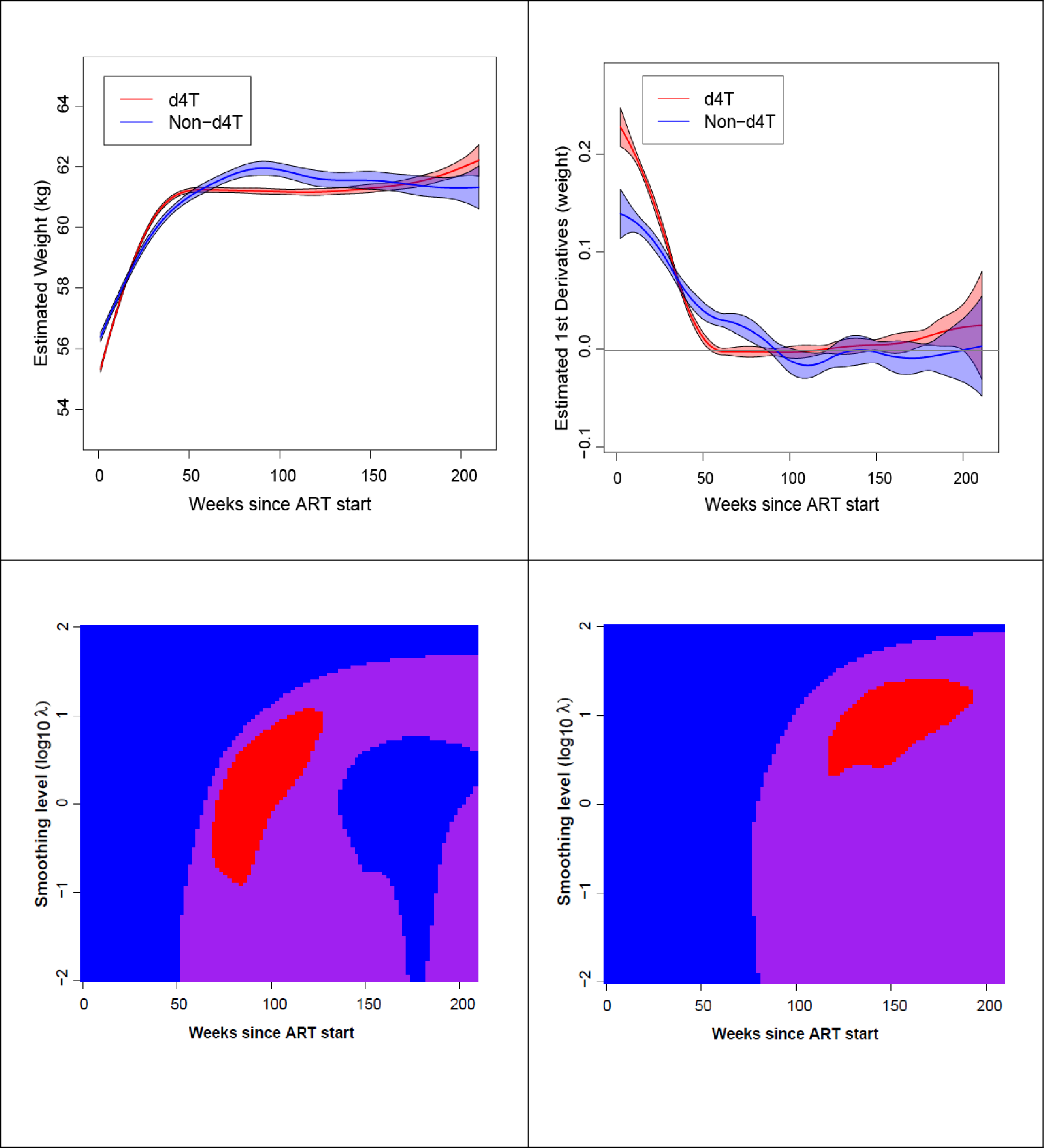

Supplement: S1 Fig — (TIF) [file pone.0220165.s001.tif]

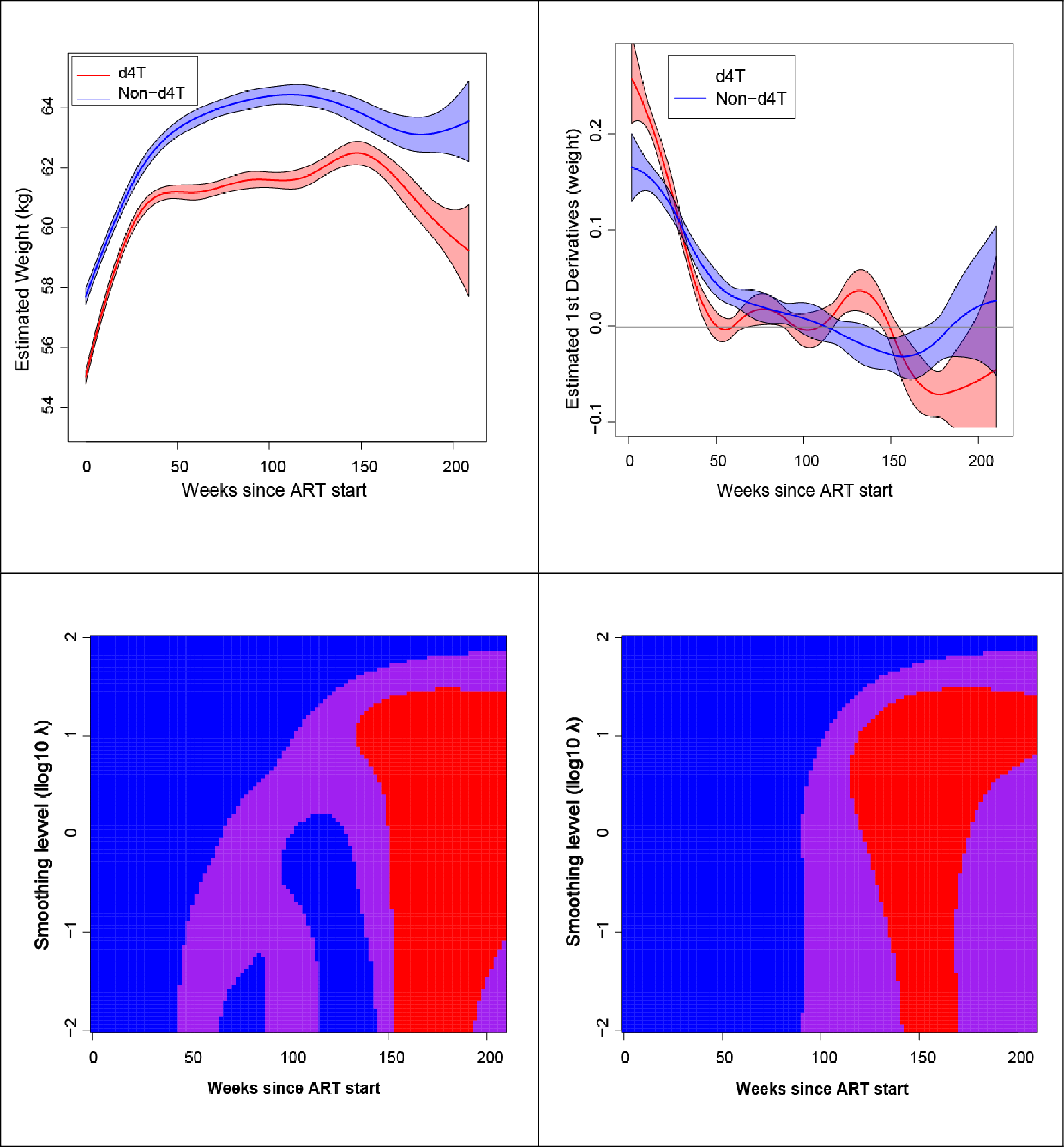

Supplement: S2 Fig — (TIF) [file pone.0220165.s002.tif]

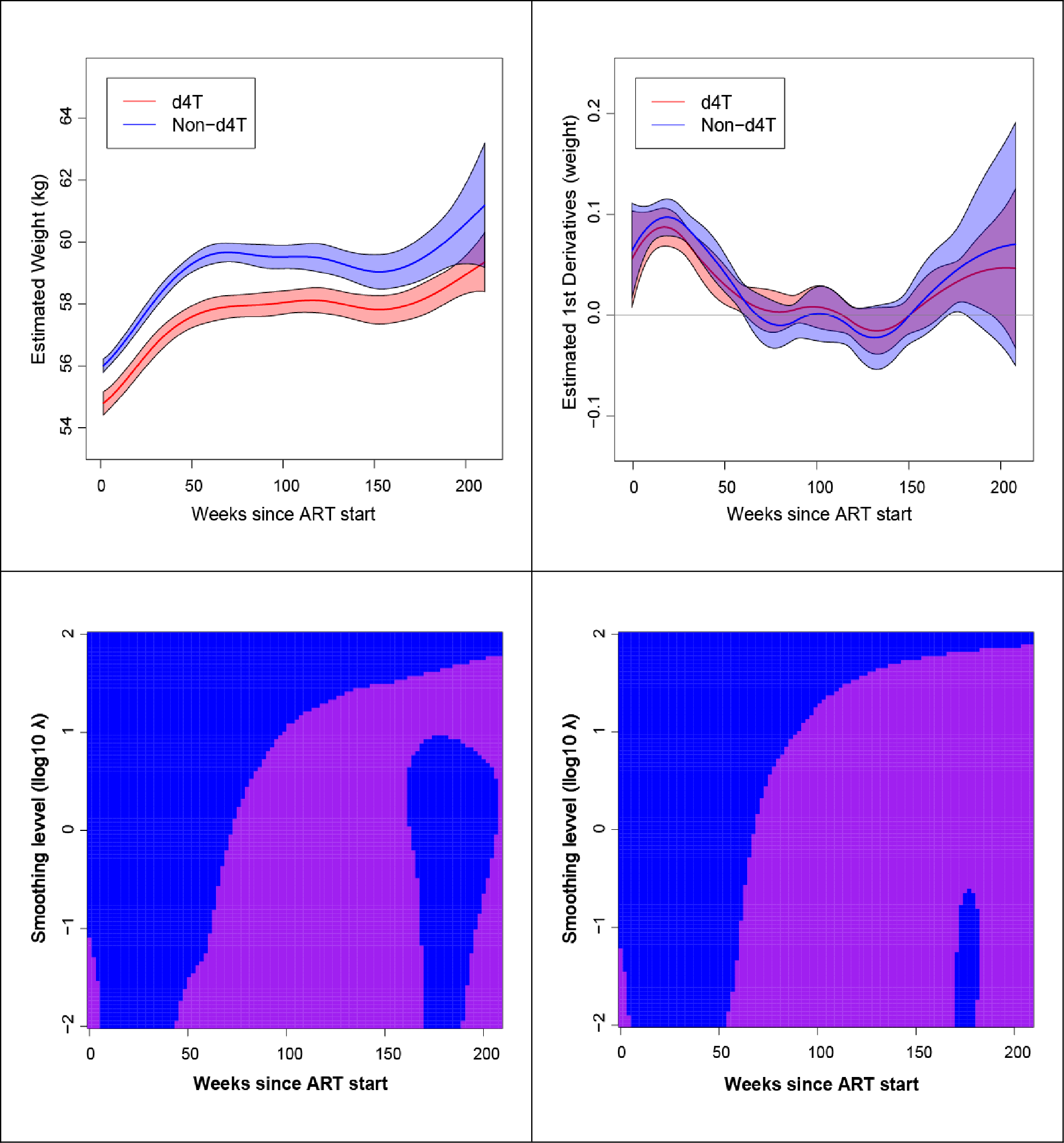

Supplement: S3 Fig — (TIF) [file pone.0220165.s003.tif]

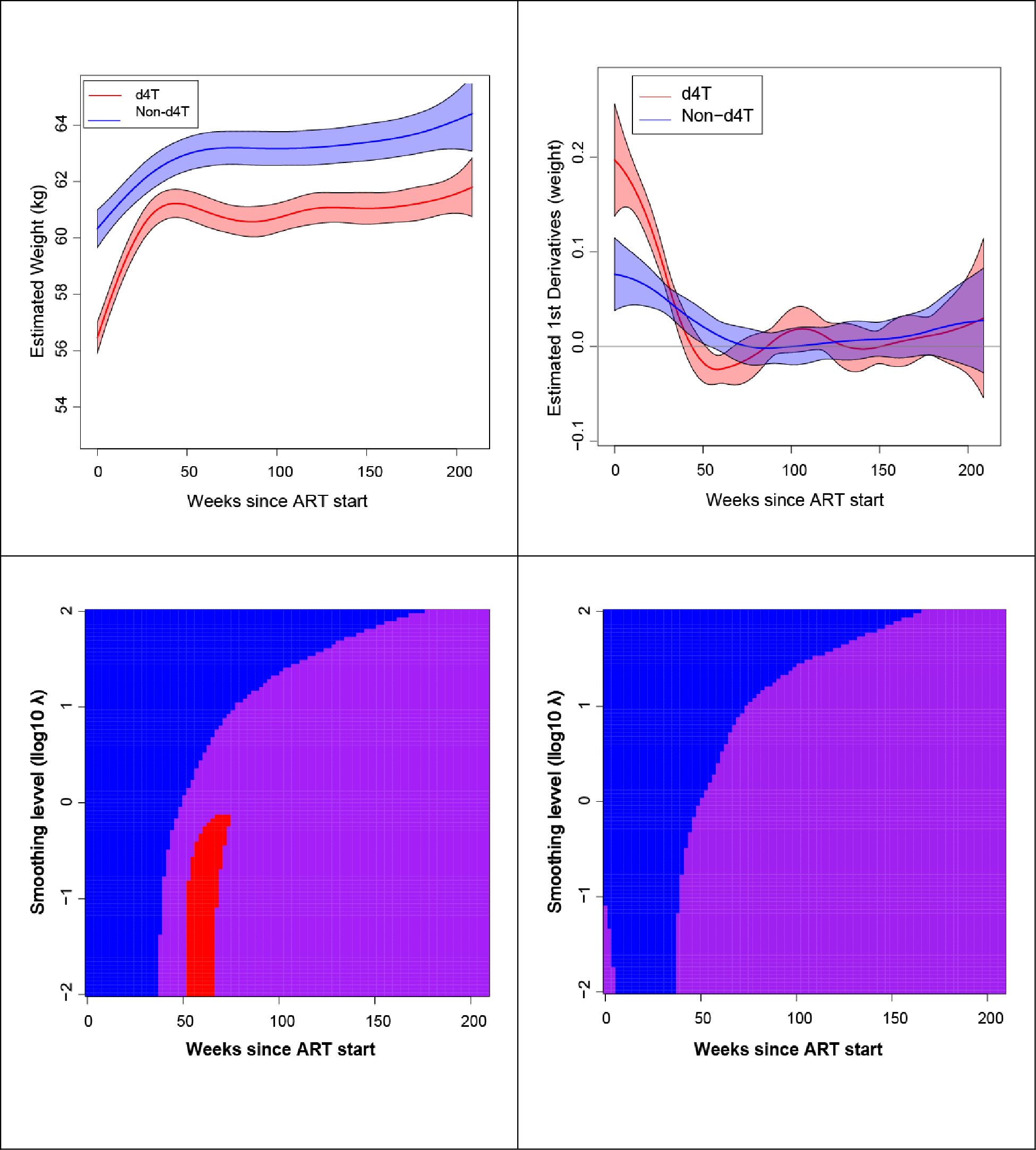

Supplement: S4 Fig — (TIF) [file pone.0220165.s004.tif]
