# Supplementary material for: PS-SiZer map to investigate significant features of body-weight profile changes in HIV infected patients in the IeDEA Collaboration
Source: PLoS One. 2020 May 1;15(5):e0220165. doi: 10.1371/journal.pone.0220165 (PMC7194369; doi:10.1371/journal.pone.0220165)
Supplement: Attachment — Submitted filename: PS-SiZer_ResponsesToTheReviewers.docx [file pone.0220165.s005.docx]

**Responses to the reviewers**

**Reviewer #1**

1. In Table 1, the authors show that PS-SiZer detect a much higher fraction of features than the other two methods. In Figure 2, the authors show that PS-SiZer can detect all features in the simulated study. In Figure 3, the estimated time to reach the plateau is much smaller in PS-SiZer than in other two methods. Taking these results together, is PS-SiZer overly sensitive? If not, can the authors perform another simulation to prove that?

**Response**: Simulations were set up to provide some *a priori* known features in the simulated data set and then use a variety of SiZer methods to discover which methods are advantageous at capturing the known features in the data. We were pleased that our simulations showed that the proposed penalized SiZer methodology picked the **correct** number of these extant features, while the other existing variants of the SiZer methodology tended to pick a wrong number of these features. Our simulation studies cover a wide range of realistic settings, consistently showing that the proposed PS-SiZer is sensitive to discovering the right number of features. Thus we are fairly convinced that PS-SiZer, particularly as we vary the smoothing levels, would not result in false positive feature detection (i.e., detecting features which are not there).

2. The authors need to increase the resolution of figure 1a and 1b, and make figure 1c axis ticks bigger. There are also some typos, e.g. in page 10 "a family of smoonth".

**Response:** Done

**Reviewer #2**

1. Please clarify what Effective Degrees of Freedom (EDF) is and the purpose.

**Response:** We have clarified that *effective degrees of freedom* encapsulate the complexity of the model as the actual *degrees of freedom* are not defined for the semiparametric models. We use the established method for their estimation, i.e. the trace of the smoother matrix (see Hastie & Tibshirani (1990) as cited in Chauduri & Marron (1999, pp. 812)).

2. It is unclear how the model addresses the irregular longitudinal data.

**Response:** We have expanded the description of the methodology used that incorporates mixed models addressing the issues of irregular visits by subjects. This is one of the major advantages of the underlying linear-mixed-model framework that we have embedded into the SiZer method.

3. The method section indicates the use of R package, mgcv::gam, as mixed model to estimate g(x) function, but data analysis used R package SiZer. Please clarify how g(x) function is estimated.

**Response:** gam() function from the R library(mgcv) is used to estimate the function g(x) at varying levels of the smoothing parameter λ.

The PS-SiZer map includes a number of levels of the smoothing parameters. In the present application, we used a range between${log10 (\lambda}_{REML})\pm2$ , where $\lambda_{REML}$ is the estimated smoothing parameter obtained via the REML approach using mixed model representation of the P-spline model. At each smoothing level, the resultant smoothing function component is obtained and extracted from the subsequent model fitting.

4. Is any difference of the proposed method vs “A Penalized Spline Approach to Functional Mixed Effects Model Analysis” by Chen and Wang (Biometrics, 2010)?

**Response:** Chen & Wang (Biometrics, 2008) proposed a new method for using the penalized spline approach for functional mixed effects models with varying coefficients. Our approach is used for the discovery of features in the underlying population regression function$.$ We expand the applicability of the SiZer approach to longitudinal designs where the P-spline model (Eilers & Marx (1996)) is used to estimate the population regression curve. Thus, the questions addressed are different.

5. Please illustrate more how the optimum smoothing parameter is estimated using the ‘Rule of thumb’ approach.

**Response:** In the revised paper we have updated the “SiZer” maps and corresponding optimum estimates to the newly developed PS-SiZer Maps. Therefore, the updated optimum smoothing parameter estimate is presented in the revised paper. In the PS-SiZer presentation the REML estimate of the mixed model is used to obtain the optimum smoothing parameter (also see above).
