# Supplementary material for: PS-SiZer map to investigate significant features of body-weight profile changes in HIV infected patients in the IeDEA Collaboration
Source: PLoS One. 2020 May 1;15(5):e0220165. doi: 10.1371/journal.pone.0220165 (PMC7194369; doi:10.1371/journal.pone.0220165)
Supplement: Attachment — Submitted filename: PS-SiZer_ResponsesToTheReviewers-with-clarification.docx [file pone.0220165.s006.docx]

**Responses to the reviewers**

**Reviewer #1**

1. In Table 1, the authors show that PS-SiZer detect a much higher fraction of features than the other two methods. In Figure 2, the authors show that PS-SiZer can detect all features in the simulated study. In Figure 3, the estimated time to reach the plateau is much smaller in PS-SiZer than in other two methods. Taking these results together, is PS-SiZer overly sensitive? If not, can the authors perform another simulation to prove that?

**Response**: Simulations were set up to provide some *a priori* known features in the simulated data set and then use a variety of SiZer methods to discover which methods are advantageous at capturing the known features in the data. We were pleased that our simulations showed that the proposed penalized SiZer methodology picked the **correct** number of these extant features, while the other existing variants of the SiZer methodology tended to pick a wrong number of these features. Our simulation studies cover a wide range of realistic settings, consistently showing that the proposed PS-SiZer is sensitive to discovering the right number of features. Thus we are fairly convinced that PS-SiZer, particularly as we vary the smoothing levels, would not result in false positive feature detection (i.e., detecting features which are not there).

No changes made in the manuscript

2. The authors need to increase the resolution of figure 1a and 1b, and make figure 1c axis ticks bigger. There are also some typos, e.g. in page 10 "a family of smoonth".

**Response:** Done

We understand and share the frustration of the reviewer. The updated figure 1c had not been attached with the uploaded revised materials by an error on our part which we regret. The figure that needed to be added is as follows:

|   (a) |   (b) |
| --- | --- |
|   (c) |   (d) |

We have updated the figure are uploading all four panels with this clarified revision.

**Reviewer #2**

1. Please clarify what Effective Degrees of Freedom (EDF) is and the purpose.

**Response:** We have clarified that *effective degrees of freedom* encapsulate the complexity of the model as the actual *degrees of freedom* are not defined for the semiparametric models. We use the established method for their estimation, i.e. the trace of the smoother matrix (see Hastie & Tibshirani (1990) as cited in Chauduri & Marron (1999, pp. 812)).

We have added this text in the document and added a comment to identify the location of the added text.

2. It is unclear how the model addresses the irregular longitudinal data.

**Response:** We have expanded the description of the methodology used that incorporates mixed models addressing the issues of irregular visits by subjects. This is one of the major advantages of the underlying linear-mixed-model framework that we have embedded into the SiZer method.

We had pointed out that our underlying model is a linear mixed effects model. It is well known that a major advantage of these models (as opposed to, say, repeated-measures models which require data to be measured at fixed times) is their ability to handle irregularly spaced longitudinal data, while modeling the covariance structure between observations. We have identified by a comment in the margin where this description is located. To avoid a full-fledged foray into mixed-model theory, we have also added the following text in this revision to explicitly address the reviewer’s comment: “As our model is a linear mixed model, a major advantage from its use is the ability to handle longitudinal data at irregularly spaced time points” and listed the book by Verbeke & Mollenbergs (2000) as an example of a reference with a description of such models.

3. The method section indicates the use of R package, mgcv::gam, as mixed model to estimate g(x) function, but data analysis used R package SiZer. Please clarify how g(x) function is estimated.

**Response:** gam() function from the R library(mgcv) is used to estimate the function g(x) at varying levels of the smoothing parameter λ. The PS-SiZer map includes a number of levels of the smoothing parameters. In the present application, we used a range between${log10 (\lambda}_{REML})\pm2$ , where $\lambda_{REML}$ is the estimated smoothing parameter obtained via the REML approach using mixed model representation of the P-spline model. At each smoothing level, the resultant smoothing function component is obtained and extracted from the subsequent model fitting.

We may have misunderstood the intent of the comment above (and, for that matter, a number of other comments). We apologize for what appears to have resulted in wasting the reviewer’s time. We thought that it was sufficient to simply clarify our intent for the benefit of the reviewer without modifying the text of the paper and thus, no changes were made in the manuscript in the previous submission. We have added the above text verbatim in the document at the end of the description of the gam() function used in this work in this submission and have identified the location of this addition by a comment in the margin.

4. Is any difference of the proposed method vs “A Penalized Spline Approach to Functional Mixed Effects Model Analysis” by Chen and Wang (Biometrics, 2010)?

**Response:** Chen & Wang (Biometrics, 2008) proposed a new method for using the penalized spline approach for functional mixed effects models with varying coefficients. Our approach is used for the discovery of features in the underlying population regression function$.$ We expand the applicability of the SiZer approach to longitudinal designs where the P-spline model (Eilers & Marx (1996)) is used to estimate the population regression curve. Thus, the questions addressed are different.

This was intended as a response to the reviewer so no changes were made in the previous version of the submitted manuscript. To ensure that we have not misunderstood the reviewer’s intent, we have included the above text verbatim in the discussion and have identified its location by comment in the margin.

5. Please illustrate more how the optimum smoothing parameter is estimated using the ‘Rule of thumb’ approach.

**Response:** In the revised paper we have updated the “SiZer” maps and corresponding optimum estimates to the newly developed PS-SiZer Maps. In the PS-SiZer presentation, the REML estimate of the mixed model is used to obtain the optimum smoothing parameter (also see above).

We have eliminated the references to the “rule of thumb” approach previously and added the above text to the submitted manuscript. In this submission, we have added a comment in the margin to identify the location of this correction.
